# The association between chiropractic integration in an Ontario community health centre and continued prescription opioid use for chronic non-cancer spinal pain: a sequential explanatory mixed methods study

**DOI:** 10.1186/s12913-022-08632-9

**Published:** 2022-11-03

**Authors:** Peter C. Emary, Amy L. Brown, Mark Oremus, Lawrence Mbuagbaw, Douglas F. Cameron, Jenna DiDonato, Jason W. Busse

**Affiliations:** 1 Department of Health Research Methods, Evidence and Impact, McMaster University, Hamilton, ON Canada; 2 Chiropractic Department, D’Youville University, Buffalo, NY USA; 3 Private Practice, 1145 Concession Road, N3H 4L5 Cambridge, ON Canada; 4 School of Public Health Sciences, University of Waterloo, Waterloo, ON Canada; 5 Biostatistics Unit, Father Sean O’Sullivan Research Centre, St. Joseph’s Healthcare- Hamilton, Hamilton, ON Canada; 6 Centre for the Development of Best Practices in Health, Yaundé, Cameroon; 7 Division of Global Health, Stellenbosch University, Stellenbosch, South Africa; 8 Department of Anesthesia, McMaster University, Hamilton, ON Canada; 9 Michael G. DeGroote National Pain Centre, McMaster University, Hamilton, ON Canada; 10 Chronic Pain Centre of Excellence for Canadian Veterans, Hamilton, ON Canada

**Keywords:** Health Services Research, Opioids, Community Health Centres, Mixed methods, Chiropractic

## Abstract

**Background:**

Emerging evidence suggests that access to chiropractic care may reduce the likelihood of initiating an opioid prescription for spinal pain; however, the impact of chiropractic care for patients already prescribed opioids is uncertain. We undertook a sequential explanatory mixed methods study to evaluate the association between initiating chiropractic care and continued opioid use among adult patients attending an Ontario community health centre (CHC) and receiving opioid therapy for chronic non-cancer spinal pain.

**Methods:**

We conducted a retrospective cohort study of 210 patient records between January 1, 2014 and December 31, 2020. We used generalized estimating equations, adjusted for patient demographics, co-morbidities, visit frequency, and calendar year, to evaluate the association between receipt versus non-receipt of chiropractic services and continued opioid use (e.g., unique opioid fills, number of refills, and dosages) up to one year following the index chiropractic visit. We also completed follow-up interviews with 14 patients and nine general practitioners from the CHC and integrated these data with our quantitative findings.

**Results:**

Over 12-month follow-up, there were lower rates of opioid fills (incidence rate ratio [IRR] = 0.66; 95% confidence interval [CI], 0.52–0.83) and refills (IRR = 0.27; 95% CI, 0.17–0.42) among chiropractic recipients (n = 49) versus non-recipients (n = 161). Although patients who did and did not receive chiropractic care began the study with the same dose of opioids, recipients were less likely to be prescribed higher-dose opioids (i.e., ≥ 50 mg morphine equivalents daily) compared to non-recipients at three months (odds ratio [OR] = 0.14; 95% CI, 0.04–0.47), six months (OR = 0.14; 95% CI, 0.05–0.40), nine months (OR = 0.19; 95% CI, 0.07–0.57), and 12 months (OR = 0.22; 95% CI, 0.08–0.62). Interviews suggested that patient self-efficacy, limited effectiveness of opioids for chronic pain, stigma regarding use of opioids, and access to chiropractic treatment were important influencing factors.

**Conclusion:**

We found that continued prescription opioid use among patients with chronic non-cancer spinal pain who received chiropractic care was lower than in patients who did not receive chiropractic care. Four themes emerged in our qualitative interviews to help provide a richer understanding of this association. Randomized controlled trials are needed to establish the effect of chiropractic care on opioid use for chronic spinal pain.

**Supplementary Information:**

The online version contains supplementary material available at 10.1186/s12913-022-08632-9.

## Background

Chronic non-cancer pain affecting the spine or other musculoskeletal tissues is a prevalent and global health problem associated with considerable socioeconomic burden. Worldwide, approximately one in five people live with chronic non-cancer pain [1–4], with seniors, women, military veterans, indigenous populations, rural inhabitants, those with lower formal education, and individuals reporting low socioeconomic status being most affected [5–7]. In Canada, the annual economic cost of chronic non-cancer pain due to medical expenditures and lost productivity was estimated between $38 and $40 billion in 2019, and this cost is expected to rise by more than 36% by the year 2030 [8]. The annual cost of chronic non-cancer pain in the United States (US) was previously estimated to be between $560 and $635 billion [9]. Opioids are commonly prescribed to patients to relieve chronic non-cancer pain, particularly in North America [10]; however, opioids provide only modest benefits [11] and are associated with important dose-dependent harms, including overdose and death [12–15]. Accordingly, governments, policy makers, and insurers have been called upon to improve support for non-opioid approaches to managing chronic non-cancer pain, particularly in vulnerable and marginalized populations [16].

Emerging evidence suggests that early access to chiropractic treatment is associated with lower initiation of opioid prescribing among patients with non-cancer spinal pain [17–21]. A 2020 systematic review and meta-analysis of six cohort studies found that patients with acute or chronic non-cancer spinal pain who received chiropractic services early in their complaint were 64% less likely than non-chiropractic users to be prescribed opioids (pooled odds ratio [OR] = 0.36; 95% confidence interval [CI], 0.30 to 0.43) [17]. A subsequent observational study of 216,504 opioid-naive patients with new-onset low back pain who received initial treatment from chiropractors versus primary care physicians had 90% lower odds of short-term opioid use (adjusted OR = 0.10; 95% CI, 0.09 to 0.10) and 78% lower odds of long-term opioid use (adjusted OR = 0.22; 95% CI, 0.18 to 0.26) [18, 19]. Similar findings have been reported by two other recent observational studies [20, 21]; however, the association between receipt of chiropractic services and continued opioid use in patients with existing opioid prescriptions is uncertain [22–24]. Moreover, previously published studies on the topic of chiropractic care and opioid prescribing have lacked in-depth, contextual understanding because they have been exclusively quantitative in nature [17–24].

To help address these knowledge gaps, we conducted a mixed methods study to evaluate the association between initiating chiropractic care and continued opioid use among adult patients with chronic non-cancer spinal pain attending an Ontario community health centre (CHC) [25, 26]. We hypothesized that younger age, male sex, health-related co-morbidities, depressive symptoms, poor health behaviours (e.g., smoking), a higher frequency of healthcare provider visits, and earlier years of our 7-year study timeframe would be positively associated with opioid use. We also hypothesized that chiropractic care would be inversely associated with opioid use [25].

## Methods

### Ethical considerations

Our study was approved by the Hamilton Integrated Research Ethics Board at McMaster University (project number 2021–10930). Approval to conduct this study was also obtained from the Chief Executive Officer at the Langs CHC [26]. All methods were carried out in accordance with the relevant guidelines and regulations and the Declaration of Helsinki.

### Study design

**Fig. 1:**
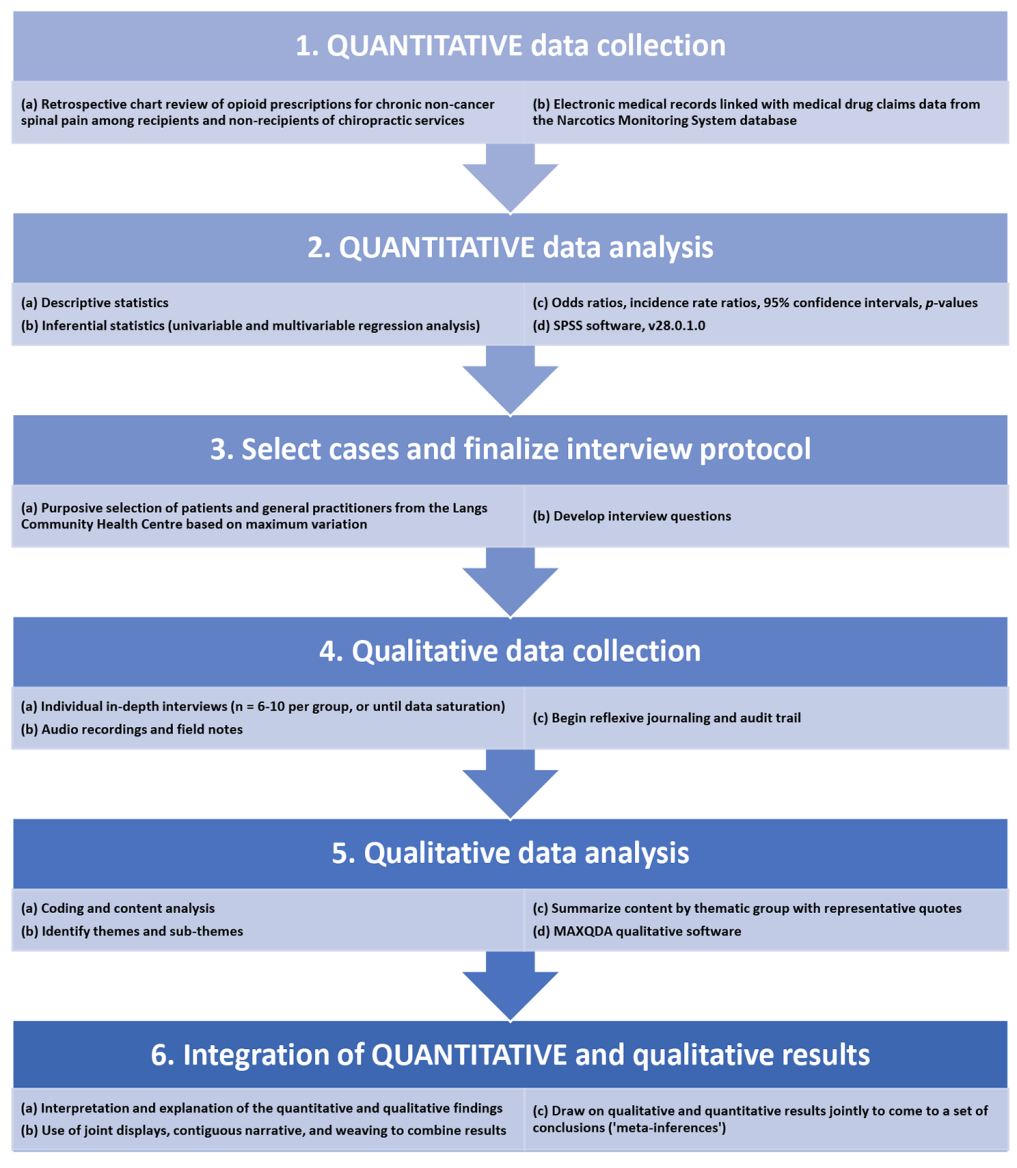
Study diagram of an explanatory sequential design of a mixed methods study on the association of chiropractic integration with opioid use for chronic non-cancer spinal pain at the Langs Community Health Centre. The quantitative and qualitative data collection and analysis phases are listed at the top of each step of the diagram. The two points of interface (or mixing) of the quantitative and qualitative phases occur in the third and final steps. The term “QUANTITATIVE” is capitalized to indicate prioritization of the quantitative phase in the study. The study procedures and outputs for each phase are listed in point-form at each step

We used a sequential explanatory mixed methods design [27]. In the quantitative phase, we obtained data via chart review [28] of electronic medical records (EMRs) of both recipients and non-recipients of chiropractic services with at least one prescribed opioid for the treatment of a chronic non-cancer spinal pain-related diagnosis at the Langs CHC [26]. In the qualitative phase, we conducted one-on-one interviews with patients and general practitioners (GPs) to explore perceptions of chiropractic integration on opioid prescribing. Complementarity [29] was our rationale for using a mixed methods approach, that is, the results from the qualitative phase of our study were used to help clarify and explain our quantitative findings. See Fig. 1 for a diagram outlining our study procedures. We followed the STROBE statement [30], the COREQ criteria [31], and the Good Reporting of A Mixed Methods Study (GRAMMS) guidelines [32, 33] for our study (Additional file 1).

### Setting

The Langs CHC is located in Cambridge, Ontario, Canada [25, 26], a medium-sized urban municipality (population: ~130,000) located 82 km southwest of Toronto. This Centre provides healthcare services to communities and vulnerable populations with high unemployment rates, multiple co-morbidities, and musculoskeletal disorders that are commonly managed with prescription opioids [25, 26]. Because chiropractic services are not publicly funded in Canada, these populations have traditionally faced barriers to accessing chiropractic care [23, 34–40]. However, since January 1, 2014 [34] a partially subsidized chiropractic spine pain program that operates on two half days per week has been offered to patients at the Centre. To be eligible to receive these services, patients have to be referred into the program by their GP. The Centre also employs a team of medical doctors, nurse practitioners, registered nurses, dieticians, social workers, community health workers, and a physiotherapist. For more complete details of the CHC’s chiropractic spine pain program, our conceptual framework, and a list of diagnostic codes used for defining our study sample, we refer readers to our study protocol [25].

### Quantitative sampling

#### Participants and data sources

We included records for all adult patients (aged ≥ 18 years) who received one or more prescriptions for opioids dispensed over a minimum period of three consecutive months, and who attended two or more appointments relating to a diagnosis of chronic spine (i.e., back or neck) pain at the Langs CHC between January 1, 2014, and December 31, 2020. The start date for quantitative sampling was January 1, 2014, which was the inaugural date of the Langs CHC’s chiropractic spine pain program [34]. Patients receiving treatment for opioid use disorder (e.g., methadone, naloxone) prior to their index visit, as well as those with spinal neoplasms or other contraindications to chiropractic treatment (i.e., fractures, infections, inflammatory arthritis, or cauda equina syndrome), were excluded from our cohort. As we were interested in patients receiving long-term opioid therapy [13], we excluded individuals who had been prescribed opioids for < 90 days at their index visit, or who did not receive any opioid fills or refills after their index visit.

We linked EMR records of all patients in our study to medical drug claims data at the Institute for Clinical Evaluative Sciences (ICES) (https://www.ices.on.ca) with their Ontario health card number. ICES is an independent, non-profit research institute whose legal status under Ontario’s health information privacy law allows for the collection and analysis of healthcare and demographic data, without consent, for health system evaluation and improvement. Patients whose health card number was incorrectly recorded in their EMR were excluded.

### Quantitative data collection

#### Variables

Opioid prescription data were obtained from the Narcotics Monitoring System database by an independent research scientist at ICES, including the number of prescribed opioid fills, the number of prescribed opioid refills (measured in 30-day equivalents), and the prescribed opioid dosage. These outcomes were measured for up to 12 months from the date of first opioid prescription following a patient’s index visit for chronic non-cancer spinal pain. To maintain temporality, the index visit for patients who received chiropractic care was their first chiropractic visit. Other variables that were extracted from the EMR included socio-demographics (age and sex), general health (smoking status and body mass index), co-morbidities (depression, anxiety, fibromyalgia, diabetes, and cardiovascular disease), and the total number of healthcare (i.e., GP or chiropractic) visits. These variables have been shown to be associated with opioid use [22, 41–48]. To increase the reliability of data extraction [28], an independent information technology specialist, who was blinded to the research questions, extracted all patient data directly from the Langs EMR database [25].

### Quantitative data analysis

#### Statistical methods

Baseline characteristics were compared between the exposed (receipt of chiropractic care) and non-exposed groups using the chi-squared test for categorical variables (or Fisher’s exact test if there was a cell frequency of < 5) and the Mann-Whitney U test for skewed continuous variables. We used generalized estimating equations (GEEs) to explore the association between exposure to chiropractic care and opioid prescribing [49, 50]. To account for potential data clustering within-subjects or between medical or chiropractic practitioners, we used a robust variance estimator to compute the standard errors for our coefficient estimates. We also conducted sensitivity analyses with different working correlation structures, including independent, autoregressive, and unstructured matrices [49, 50]. The specified link function in our GEE models was based on the data distribution (e.g., log-linear for data fitting a Poisson distribution, binomial for binary data).

We used GEEs with a Poisson distribution when the outcomes were counts (i.e., total number of unique opioid fills and subsequent refills over the entire course of follow-up, tabulated at the end of follow-up). We estimated incidence rate ratios (IRRs) for differences between the chiropractic and non-chiropractic groups using Poisson log-linear GEEs and reported the associated 95% CIs and *p*-values.

We used GEEs with a binomial distribution when the outcome was opioid dosage. We assessed opioid dosages at 90-day intervals, dichotomized into higher (≥ 50 mg) morphine equivalents daily (MED) or lower (< 50 mg) MED [11] and compared these between the chiropractic and non-chiropractic groups from baseline to 12-month follow-up. We originally planned to dichotomize opioid dose using a different threshold (≥ 90 mg versus < 90 mg MED) [25], but we modified our approach to reflect the central tendency of MED in our patient sample. We estimated between-group differences for dosage using a binary logistic GEE and reported these with ORs, 95% CIs, and *p*-values. To calculate the MED for each prescribed opioid, we multiplied the quantity × the milligrams per unit dispensed × drug-specific conversion factors (Additional file 2) [11, 13].

#### Quantitative variables and study size

For each outcome of interest, we built univariable and multivariable models to estimate the crude and adjusted associations, respectively, between patients that did or did not receive chiropractic care (1 = received; 0 = did not receive) and opioid use. We grouped covariates into blocks (e.g., socio-demographic, health-related, depressive symptoms, health behaviours, and healthcare visits) and these were sequentially entered into our models, with time (i.e., calendar year) as an additional covariate and chiropractic/non-chiropractic care as the main exposure variable. To guard against over-fitting of our regression models [51], we set a minimum threshold of 10 events per category for each independent variable (i.e., minimum sample of 150 patient records) to ensure that each variable had sufficient discriminant power to detect an association with opioid use, if an association existed.

We assessed model fit using the quasi-likelihood under the independence model criterion (QIC) [50, 52]. Correlation structures with the lowest QIC scores (closest to zero) were judged as the best model fit for the data. We also explored variance inflation factors (VIFs) to assess collinearity between independent variables. If multicollinearity was detected between two or more variables (i.e., VIFs ≥ 5) [53], we compared regression models, each separately containing one of the collinear variables, to one another and selected the model containing the variable that produced the lowest Akaike information criterion (AIC) value. The two-sided statistical significance level ($\alpha$) for all quantitative analyses was 5%, and all data and comparative analyses were performed using SPSS v28.0.1.0 (IBM SPSS Statistics).

### Qualitative sampling

For the qualitative phase of our study, we used stratified purposive sampling to select a sub-sample of chiropractic and non-chiropractic patients, whose charts we examined in the quantitative phase, to participate in one-on-one interviews [54]. This was the first stage of integration between our quantitative and qualitative study phases [55]. We also recruited a purposive sample of GPs from the Langs CHC. The lead author (PCE) conducted recruitment via telephone or e-mail using participant contact information provided by the Langs CHC administration. We offered gift cards ($10 for GPs, $30 for patients) as incentives for participation. We used maximum variation [54] in choosing participants, based on age, sex, and the number of years attending the CHC (for patients) or years in practice (for GPs), to encourage a range of sociodemographic characteristics and perspectives. We also collected patients’ primary spine pain complaint and current opioid dose. We aimed to interview a minimum of 12–20 patients and 6–10 GPs [54], with interviews continuing until saturation; the point at which no new information was obtained from participants in the GP, chiropractic, and non-chiropractic groups [56]. We used fundamental qualitative description [56, 57] as our methodological orientation to underpin the qualitative phase of our study.

### Qualitative data collection

The lead author (PCE), a health research methodologist with expertise in mixed methods and qualitative research, conducted one-on-one (individual) semi-structured interviews with participants. Interviews were conducted either virtually (n = 3) using the Zoom videoconferencing application (Zoom Video Communications, Inc.) or in-person (n = 20), based on participant preference. We promoted confidentiality by conducting the interviews in a private office separate from the medical clinic at the Langs CHC. We obtained informed consent from participants before the start of each interview. Five members of our research team (PCE, ALB, MO, LM, JWB) developed the patient and GP interview guides (see Additional files 3 and 4, respectively) based on relevant literature [17–24, 27] and our quantitative findings. Three of the five members (PCE, ALB, JWB) also have content expertise in the subject area of our study.

We audio recorded virtual interviews using Zoom’s built-in recording feature and in-person interviews using MacIntosh recording software (Audio Recorder v1.3, FIPLAB Ltd.). The lead author (PCE) also took field notes after each interview to document other observations and emergent themes. To promote trustworthiness in our qualitative data, we employed member-checking [27] by sending the raw transcripts and a summary of our results to participants for feedback or correction. We also kept an audit trail of our qualitative data collection and analysis procedures [56]. A summary of our investigator reflexivity is provided in Additional file 5.

### Qualitative data analysis

We transferred all interview audio recordings into the software program, MAXQDA (http://www.maxqda.com), and the lead author (PCE) transcribed the audio recordings verbatim. After participant identifiers were removed, another member of the research team (JD) reviewed a random sample of 15% of the transcripts for accuracy and found only a few minor typographical errors. All transcripts were then independently coded by two investigators (PCE, ALB) using an inductive content analytic approach [56]. The aim of this strategy was to descriptively summarize the information to ensure the ‘best fit to the data’ [57]. We used both open and axial coding in our data analysis: open coding to develop concepts from the data, and axial coding to relate these codes (or concepts) to one another followed by the identification of themes, sub-themes and representative quotes [27].

The two investigators undertaking coding of transcripts met three times throughout the analysis (i.e., after every seven to eight interviews) to compare themes and arrive at a final, agreed-upon set of themes through discussion. We organized these themes into tabular form and selected representative quotations for each theme/sub-theme [27]. We created joint display tables as part of our data integration procedures (Fig. 1), and our qualitative and quantitative results were further combined using contiguous narrative and weaving approaches [27, 55]. We then drew upon our qualitative and quantitative results jointly to come to a set of conclusions (i.e., ‘meta-inferences’) [27].

## Results

### Quantitative findings

**Fig. 2:**
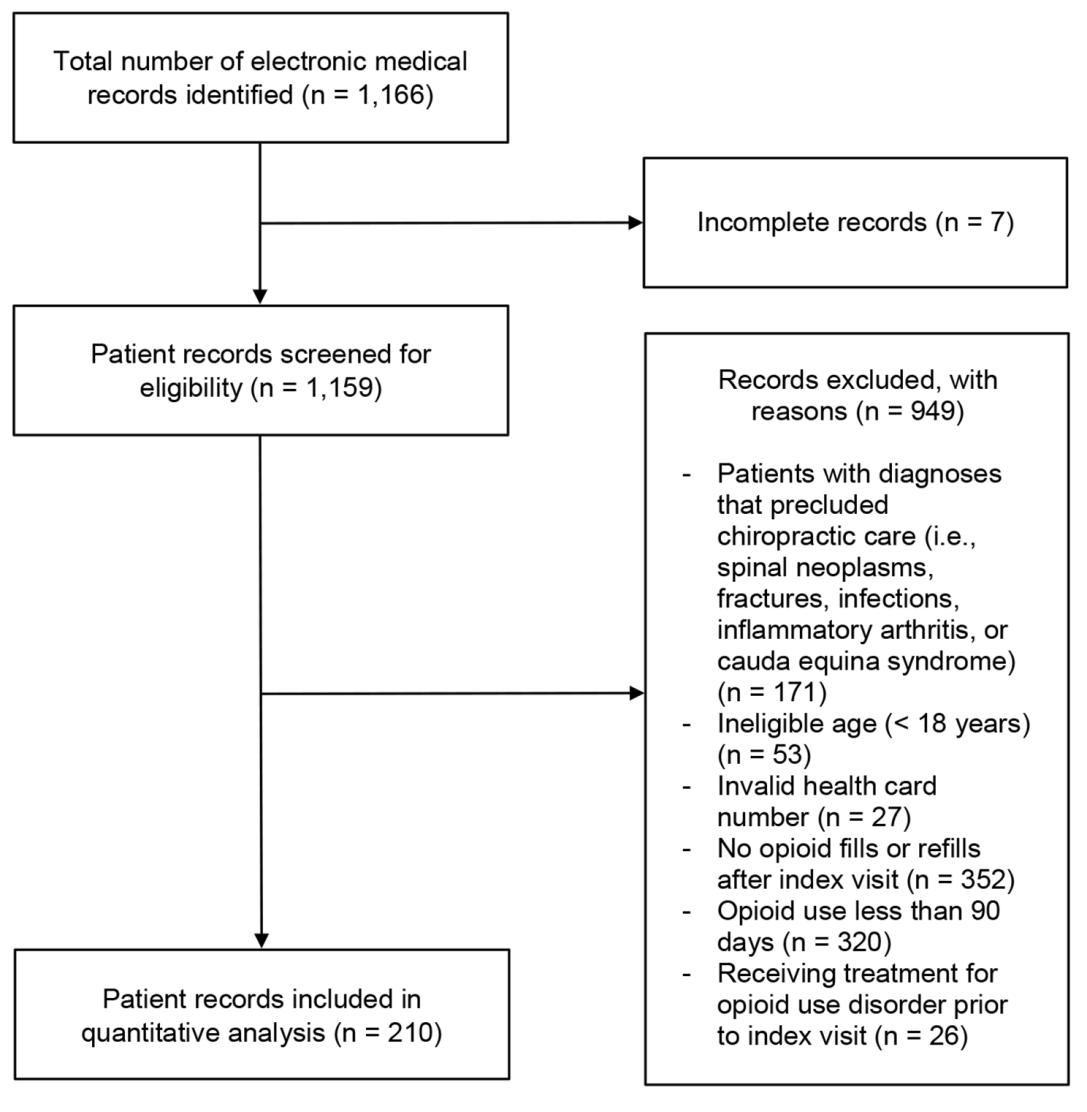
Flowchart of cohort inclusion for the quantitative analysis

We identified a total of 1,166 patient records, and 210 met eligibility criteria for inclusion in our quantitative analysis (Fig. 2).

#### Cohort characteristics

The majority (70%) of patients were ≥ 45 years of age, over half (58%) were female, approximately one-third (36%) were smokers, and 18% were obese. Patients presented with high rates of co-morbid conditions including cardiovascular disease (65%), depression (55%), anxiety (42%), diabetes (29%), and fibromyalgia (11%). The median number of healthcare visits per patient over 12 months was 5 (inter-quartile range [IQR], 2 to 8), and 23% received chiropractic services. In terms of opioid use, the median number of unique opioid fills over 12-month follow-up was 2 (IQR, 1 to 2), the median number of 30-day (or equivalent) opioid refills was 4 (IQR, 1 to 12), and baseline opioid dosage ranged from 2 to 840 mg MED (median = 30; IQR, 15 to 67 mg MED). Chiropractic recipients had similar baseline characteristics to those who did not receive chiropractic services (Additional file 6).

#### Quantitative analysis

In our adjusted regression analysis, we found inverse associations between receipt of chiropractic care and filling an opioid prescription (IRR = 0.66; 95% CI, 0.52 to 0.83) or refilling an opioid prescription (IRR = 0.27; 95% CI, 0.17 to 0.42) (Table 1). There was no difference in the odds of being prescribed a higher dose of opioids (i.e., ≥ 50 mg MED) between chiropractic recipients and non-recipients at baseline (OR = 0.61; 95% CI, 0.26 to 1.47); however, chiropractic recipients were less likely to receive a higher opioid dose compared to non-recipients at three months (OR = 0.14; 95% CI, 0.04 to 0.47), six months (OR = 0.14; 95% CI, 0.05 to 0.40), nine months (OR = 0.19; 95% CI, 0.07 to 0.57), and 12 months (OR = 0.22; 95% CI, 0.08 to 0.62). At 12-month follow-up, 29 of 49 (59%) chiropractic recipients had discontinued using opioids compared to 50 of 161 (31%) non-recipients.

**Table 1:** Unadjusted and adjusted effect sizes of the outcomes for prescription opioid use among recipients (n = 49) and non-recipients (n = 161) of chiropractic services treated for chronic non-cancer spinal pain at the Langs Community Health Centre between January 1, 2014, and December 31, 2020

Outcome measure	Univariable	***P***-value	Multivariable ^a^	***P***-value
	**Effect size (95% CI)**		**Effect size (95% CI)**	
Opioid fills ^b^	0.69 (0.56 to 0.85)	< 0.001	0.66 (0.52 to 0.83)	< 0.001
Opioid refills ^c^	0.38 (0.24 to 0.60)	< 0.001	0.27 (0.17 to 0.42)	< 0.001
Opioid dosages ^d^ • Baseline • 3 months • 6 months • 9 months • 12 months	0.77 (0.38 to 1.55) 0.42 (0.18 to 0.99) 0.33 (0.13 to 0.82) 0.39 (0.17 to 0.94) 0.52 (0.23 to 1.19)	0.466 0.049 0.018 0.035 0.123	0.61 (0.26 to 1.47) 0.14 (0.04 to 0.47) 0.14 (0.05 to 0.40) 0.19 (0.07 to 0.57) 0.22 (0.08 to 0.62)	0.270 0.001 < 0.001 0.003 0.004

*CI* confidence interval

^a^ Adjusted for age, sex, smoking status, obesity, depression, anxiety, fibromyalgia, diabetes, cardiovascular disease, visit frequency, and calendar year

^b^ Opioid prescription fills over 12-month follow-up. An incidence rate ratio < 1 indicates a lower rate of opioid fills in the recipient group

^c^ Opioid prescription refills (of 30 days or equivalent) over 12-month follow-up. An incidence rate ratio < 1 indicates a lower rate of opioid refills in the recipient group

^d^ Opioid dosage over 12-month follow-up. An odds ratio < 1 indicates a reduced likelihood of higher opioid dosage (i.e., ≥ 50 mg morphine equivalents daily) in the recipient group

Patients with an index visit date in a more recent calendar year also had a lower rate of opioid refills (IRR = 0.82; 95% CI, 0.73 to 0.93) and were less likely to be receiving higher dose opioids at three months (OR = 0.73; 95% CI, 0.57 to 0.94) and six months (OR = 0.78; 95% CI, 0.62 to 0.99) (Additional file 7 [b, d, e]). Those with a higher frequency of healthcare visits were more likely to have a higher rate of opioid refills (IRR = 1.06; 95% CI, 1.02 to 1.09) and to be receiving higher dose opioids at three months (OR = 1.11; 95% CI, 1.02 to 1.21), six months (OR = 1.09; 95% CI, 1.01 to 1.18), nine months (OR = 1.10; 95% CI, 1.02 to 1.19), and 12 months (OR = 1.12; 95% CI, 1.03 to 1.21) (Additional file 7 [b, d-g]). Male sex, depression, and fibromyalgia were positively associated with opioid dosage at various time points (Additional file 7 [c-e]). Contrary to our predictions, anxiety and obesity were negatively associated with opioid dosage (Additional file 7 [c, d, f]), while younger age was not associated with opioid use in our patient sample (Additional file 7). All VIFs were less than 1.4, suggesting no important multicollinearity among independent variables.

### Qualitative and mixed methods findings

Twenty-three patients were recruited for interviews and 14 participated. Five patients scheduled interviews but cancelled (two chiropractic recipients, three non-recipients), two scheduled interviews but did not attend (one recipient, one non-recipient), one declined for health reasons and one was not interested. Of those who were interviewed, eight were chiropractic recipients and six were non-recipients. Among GPs, four of six medical doctors and five of six nurse practitioners completed interviews. Two medical doctors declined participation because of lack of time, and one nurse practitioner expressed interest but did not respond to further interview requests. In total, 23 interviews were completed (14 patients, nine GPs). The median durations of interviews were 25 min (range, 19 to 56) for patients and 38 min (range, 20 to 40) for GPs.

The majority (79%) of the 14 patients we interviewed were female, most (86%) were either receiving disability benefits or were unemployed, and the majority (71%) had previously received at least one opioid prescription for chronic non-cancer spinal pain. The median dosage for those currently receiving opioid medications was 19 mg MED (range, 14 to 90). Among patients and GPs, there was a large range of ages (33 to 82) and number of years attending the Langs CHC (patients: 2 to 43) or years in practice (GPs: 1 to 26), demonstrating variability among participants (Additional file 8).

Among all 23 participants, one non-chiropractic patient and four GPs made minor revisions to clarify statements from their interviews during member-checking. No other participants requested content changes or corrections to their transcripts or results. We determined that data saturation had been reached when only two new codes emerged from chiropractic recipient interviews 6, 7 and 8 (with no new codes from interviews 7 and 8); only one new code emerged from non-recipient interview 4 (with no new codes from interviews 5 and 6); and only one new code emerged from GP interviews 7, 8 and 9. At this point, participant recruitment was concluded.

#### Coding tree

We identified 37 codes across interviews which were categorized into four major themes: (1) patient self-efficacy, (2) accessibility of non-pharmacological services, (3) stigma regarding use of opioids, and (4) impact of treatment. Codes pertaining to patient self-efficacy were stratified into two sub-themes, ‘active versus passive approaches’ and ‘resistance to taking medication.’ This latter sub-theme was common among chiropractic patients. For our second theme, we created the sub-themes ‘lack of access to non-pharmacological treatment options’ and ‘access to chiropractic services at the Langs CHC.’ Lack of access to non-pharmacological services (e.g., chiropractic, physiotherapy) was identified in nearly all (21 of 23) participant interviews and was reported as a common facilitator of opioid use. Our third theme captured codes related to the opioid crisis such as negative media coverage or lived experiences. Some patients also expressed a sense of judgement from others for using prescription opioids. The remaining codes related to patients’ or GPs’ perspectives on the impact of treatment for chronic non-cancer spinal pain, including sub-themes of pain relief, functionality, recognition of the limited effectiveness of opioids for chronic pain, and anxiety and fear surrounding opioid withdrawal. Descriptions and frequency counts of each of our major themes, sub-themes, and representative participant quotes are provided in Additional file 9. Our main quantitative findings are presented with qualitative data as joint displays in Tables 2 and 3.

**Table 2:** Joint display of the quantitative association between receipt of chiropractic services at the Langs Community Health Centre and prescription opioid use, representative qualitative interview quotes, and meta-inferences

Variable	Quantitative results	Qualitative interview quotes	Meta-inferences
Receipt of chiropractic care (n = 49)	• Negative association with total number of opioid fills (adjusted IRR = 0.66) • Negative association with total number of opioid refills ^a^ (adjusted IRR = 0.27) • Negative association with higher opioid dosage at: 3-month follow-up (adjusted OR = 0.14) 6-month follow-up (adjusted OR = 0.14) 9-month follow-up (adjusted OR = 0.19) 12-month follow-up (adjusted OR = 0.22)	Resistance to taking medication: • *“I don’t want to take so many medicine[s]. … It’s too much chemical going in your body, it’s no good. … I try to take, even with my pain, [only] one Tylenol #3, and [then] I will take Advil or extra strength Aspirin or Tylenol every six hours [for the rest of the day].”* DC Patient 4 • *“I try and adhere against [taking] Tylenol #3, if I can help it.”* DC Patient 5 • *“I’ve been prescribed [opioids], … but I just started reading about stuff, what it does to your liver and what it does to other organs in your body. I just, I chose other methods, i.e., like chiropractic, massage, I bought a hot tub – hydrotherapy. Just stuff like that. … I’m just so not a drug guy.”* DC Patient 6 Impact of chiropractic treatment on chronic spinal pain: • *“When I first started coming [to see the chiropractors at Langs] I couldn’t hardly walk and get in my car, to get in and out of the car, it was a challenge. And after a few chiropractor treatments, it got much better. And some days I couldn’t even turn my head sideways to see driving the car, and that got fixed. It’s gone well. Sometimes, it comes back a little bit, but then I just think – now I can get this fixed with the chiropractor.”* DC Patient 3 • *“When I had the chiropractor [treatments], … it wasn’t just the treatment, it was [them] givin’ me ideas of things to do to help yourself. And those kind[s] of things are so valuable.”* DC Patient 5 • *“It really brings home this message of – a chemical going into your body is only one way to influence this. So, if somebody’s having a positive experience [with chiropractic treatment], and we have had lots of people who’ve had positive experiences, it can mean the difference between not increasing a dose [versus increasing a dose]. Not starting a dose? I would say that there probably are situations where we’ve had that as well.”* GP 7 Access to chiropractic services at the Langs CHC: • *“A lot of our patients are from low income [backgrounds] and have transportation issues. So, having [chiropractic] services available for them here is very important.”* GP 2 • *“I just didn’t have the funds to have chiropractic [treatment]. But then when it was offered to me at Langs, I was just like – yeah, I’ll take it!”* DC Patient 6 • *“We definitely need those added services [for patients] who have chronic pain because it’s an option. … We need some way of getting that patient to treat pain in non-drug ways.”* GP 3	The rate of filling and refilling opioid prescriptions was 34% and 73% lower, respectively, among chiropractic recipients versus non-recipients. Over 12 months of follow-up, chiropractic recipients were also between 78% and 86% less likely than non-recipients to have received a higher (≥ 50 mg MED) opioid dose. Patients who were referred by their GP for chiropractic services at Langs may have been more resistant to taking opioids than patients who were not referred for chiropractic services. Access to chiropractic treatment also gave patients and their GPs another non-opioid pain management option.

*CHC* community health centre, *DC* doctor of chiropractic, *GP* general practitioner, *IRR* incidence rate ratio, *MED* morphine equivalents daily, *OR* odds ratio

^a^ Prescription opioid refills were measured in 30-day equivalents

**Table 3:** Joint display of the quantitative associations of visit frequency and calendar year with prescription opioid use at the Langs Community Health Centre, representative qualitative interview quotes, and meta-inferences

Variable	Quantitative results	Qualitative interview quotes	Meta-inferences
Higher frequency of healthcare visits (n = 210) ^a^	• Positive association with total number of opioid refills ^b^ (adjusted IRR = 1.06) • Positive association with higher opioid dosage at: 3-month follow-up (adjusted OR = 1.11) 6-month follow-up (adjusted OR = 1.09) 9-month follow-up (adjusted OR = 1.10) 12-month follow-up (adjusted OR = 1.12)	Passive pain management strategies: • *“I found, like, after I’d been in [for chiropractic treatment] on a Tuesday and they’d put me all back in shape again, and put my shoulder back in, I felt great by Thursday. Thursday it was time to come back in. So, it kept me even. It kept the pain down. … With me comin’ in twice a week, I knew at least for four days out of the week I was going to be fine.”* DC Patient 7 • *“You expect the doctor to fix it, ‘cause that’s how we were brought up.”* Non-DC Patient 6 • *“Some of our people are just rather passive in their approach to their care.”* GP 1 • *“Everything is short-term. [My pain is] chronic. It’s there to stay because I try everything. … I’ve tried physio, chiro, … I even have steroid needles [at the] pain clinic, … and saw a sport therapist person [physiatrist] for a different type of needle [epidural injection]. … I take the Robaxacet if I’m in too much pain, or Advil. … They gave me Percocet. … [Even with regular] massage therapy [and] osteopathy, I go to bed and the day after and it’s still there. … I wish somebody could go inside and just fix [it]. It’s just a hard place to be fixed, it’s not made to be fixed – the back.”* Non-DC Patient 2	Patients with a higher frequency of healthcare visits had a higher rate of refilling opioid prescriptions and were more likely to be receiving higher dose (≥ 50 mg MED) opioids over 12-month follow-up. Patients who relied on passive pain management strategies may have been more likely to visit their healthcare providers more often and obtain opioid prescriptions on a more frequent basis and at higher doses.
Index visit in more recent calendar year (n = 210)	• Negative association with total number of opioid refills ^b^ (adjusted IRR = 0.82) • Negative association with higher opioid dosage at: 3-month follow-up (adjusted OR = 0.73) 6-month follow-up (adjusted OR = 0.78)	Reduced opioid prescribing in recent years: • *“When I graduated [from medical school] in 1996, the overwhelming message to us was that we weren’t treating pain adequately; we weren’t treating it aggressively enough. And then of course, OxyContin was just the ‘new guy’ on the block and all that was wonderful and there was no ceiling dose, and you know the rest of that story.”* GP 7 • *“In the last four or five years [here at Langs], we’ve worked even harder at getting people off opioids.”* GP 3 • *“The goal is that opioids are not used for chronic non-cancer pain. I think over the last five [or] 10 years we’ve seen [a] reduction in use, and a lot of patients have been titrated down in their doses and are using more appropriate [levels of opioid] medications now.”* GP 8 • *“Having followed the sort of structure that we normally do here now in the last five years [with opioid prescribing], there’s much fewer people on [high doses].”* GP 3	Patients whose index visit date was in a more recent calendar year had a lower rate of refilling opioid prescriptions and were less likely to be receiving higher dose (≥ 50 mg MED) opioids at 3- and 6-month follow-up. GPs at Langs have made a concerted effort in recent years to reduce opioid prescribing.

*DC* doctor of chiropractic, *GP* general practitioner, *IRR* incidence rate ratio, *MED* morphine equivalents daily, *OR* odds ratio

^a^ Healthcare visits constitute GP and chiropractic visits

^b^ Prescription opioid refills were measured in 30-day equivalents

## Discussion

Among patients receiving long-term opioid therapy for chronic non-cancer spinal pain, we found that initiating chiropractic care was associated with fewer fills and refills for prescription opioids and, when prescribed, reduced dosage of opioids. Based on our qualitative findings, use of opioids was influenced by patients’ self-efficacy and concerns about opioid-related harms, recognition of the limited effect that opioids may have on chronic pain, increasing stigma regarding use of opioids, and access to non-pharmacological treatment options.

Our findings are supported by other uncontrolled observational studies [22–24]. A retrospective analysis of quality assurance data from a CHC in Manitoba, Canada [23] found that patients referred for chiropractic services had a 22% decrease in the number of opioid tablets used after attending an average of five chiropractic visits. Between baseline and discharge, the number of chiropractic patients prescribed opioids within this health care centre decreased 26% [23]. Findings of reduced opioid usage among patients receiving chiropractic services in US Veteran Administration [22] and CHC [24] clinic settings have also been recently reported.

The integration of quantitative and qualitative methods in our study generated several insights into our results. As highlighted in our interviews, patients who were referred for chiropractic services at the Langs CHC may have been more resistant to taking opioid medication than patients not referred for chiropractic services, a sentiment supported by some published evidence [58]. In addition, GPs indicated that access to chiropractic treatment gave them another non-opioid pain management option. Lack of access to non-pharmacological services (e.g., chiropractic, physiotherapy) was reported by several participants as a facilitator of opioid use, while chiropractic patients and GPs identified negative stigma associated with the use of opioids as a common barrier. We also found in our cohort that the proportion of chiropractic recipients who discontinued using opioids was nearly double that of non-recipients. These factors may help explain why chiropractic recipients obtained fewer opioid prescriptions and were less likely to be receiving higher opioid doses up to one year after presentation.

Similar to previous research [42, 44], we found that a higher frequency of healthcare visits was positively associated with opioid use. Patients with lower self-efficacy or experiencing greater difficulty coping with their pain may have been more likely to visit their healthcare providers more often and obtain opioid prescriptions on a more frequent basis and at higher doses. Recent evidence suggests that active pain self-management programs that include exercise, goal setting, education, and counselling on opioid discontinuation, as well as interventions aimed at supporting prescribers’ adherence to guidelines (e.g., chart audits, tracked performance metrics related to high-dose prescribing), can increase the likelihood of patients reducing their opioid dose or discontinuing opioid treatment [59]. However, as was frequently mentioned by both GPs and patients in our interviews (see Theme #2 in Additional file 8), accessibility of non-pharmacological treatment options remains a challenge, particularly for persons who are unemployed or from low income backgrounds [26, 34–40, 42–44, 59].

We found that patients with an index visit date in a more recent calendar year had fewer opioid prescription refills and were less likely to receive higher opioid doses at 3- and 6-month follow-up. Current guidelines [13, 60] recommend optimization of non-opioid and non-pharmacologic treatments prior to opioid use, while limiting opioid doses (when first used with patients) to less than 50 mg MED, and offering a trial of voluntary tapering if doses are already ≥ 90 mg MED. Accordingly, several GPs indicated in their interviews that a concerted effort, in the form of internal chart audits and clinical team meetings, had been made in recent years to reduce opioid prescribing at the Langs CHC. When controlling for calendar year in our analyses, however, we found that the number of opioid fills, refills, and dosages were still considerably lower among chiropractic recipients.

Several observational studies have reported an association between use of chiropractic services and reduced opioid prescribing [17–21, 61] or reduced opioid use [22–24]. Previous observational research [34–39] also suggests that integrating chiropractic services with physician management of spine-related pain is associated with improved patient outcomes and potential for cost savings (e.g., reductions in advanced imaging, GP visits, and specialist referrals). When accessed as a first-line treatment, chiropractic services may also help to delay, and in some cases prevent, opioid prescription [17–21, 61]. In one of our interviews (see Theme #2, first sub-theme, Additional file 8), the following GP expressed that,

*“…having access to any kind of additional modalities in a timely and efficient manner … would probably reduce the need for opioids in the first place.”* GP 9

Our findings add to a growing body of observational evidence that suggests integration of chiropractic services into primary care centres [23, 24, 34–39] and interdisciplinary spine care pathways [62] would reduce barriers to access and potentially reduce use of opioids among patients with chronic non-cancer spinal pain. However, since the efficacy of non-pharmacological interventions including chiropractic care for reducing opioid use remains uncertain [59], and observational research is susceptible to selection bias and confounding [63], well-designed randomized controlled trials are needed to confirm these findings.

Our qualitative findings suggest that lower opioid use is also related to factors such as self-efficacy and concern about opioid-related harms, access to non-pharmacological care, stigma, and knowledge of opioid effectiveness on chronic pain. Future research should investigate these factors further to inform their association with opioid use.

### Strengths and limitations

Our study has several strengths. First, we used patient health card numbers to link EMR data with medical drug claims data from the Narcotics Monitoring System database at ICES to verify patient opioid prescriptions and dosages. Second, we specified the anticipated direction of association for each independent variable in our regression models *a priori* to provide greater confidence in our findings. Third, we used GEEs to account for hierarchical clustering and to control for differences in confounding factors between our exposed (receipt of chiropractic care) and unexposed groups. To account for policy changes in opioid prescribing, we controlled for calendar year in our analyses. This helped to more clearly delineate between a reduction in opioid use associated with access to chiropractic services versus confounding by policy change. Additional strengths included limited missing data (< 1%), direct data export from the EMR to avoid extraction errors [28], and validation of our qualitative data via member-checking. A final strength of our study is our qualitative findings, which provided a richer understanding of the barriers and facilitators to opioid use and how chiropractic services may have been used by patients and GPs to reduce reliance on opioid prescribing for chronic non-cancer spinal pain.

Our study also has several limitations. Due to the retrospective design in our quantitative phase, certain variables that may be associated with opioid use were unavailable. For example, due to the constraints of data recorded in the Langs EMR, we were unable to extract information on co-interventions that patients may have received outside of the CHC, as well as baseline severity/chronicity of patients’ spine-related pain, and additional potential confounders such as employment status or other mental health and pain conditions. However, Langs CHC patients are unlikely to access private healthcare services elsewhere due to socioeconomic disadvantages [23–26, 34–40]. In addition, we used receipt of opioid prescriptions over three consecutive months, combined with multiple clinic visits for a non-cancer spinal pain diagnosis at the Langs CHC, as a proxy for chronic non-cancer spinal pain. Another limitation is that despite restricting our EMR data extraction to patient encounters related to non-cancer spinal pain, and only including opioid medications prescribed on or between these visit dates, it remains possible that opioids may have been prescribed for other indications. However, this would have attenuated the association between chiropractic care and opioid use [64]. Furthermore, our primary outcome measures (i.e., opioid prescriptions and dosages) are surrogates for patient-important outcomes such as functional improvement or pain reduction. An inherent limitation with using a sequential mixed methods design (i.e., quantitative followed by qualitative) is that 11 months elapsed between our quantitative and qualitative study phases, subsequently limiting our qualitative data collection. For instance, some individuals whom we attempted to recruit from the larger cohort were no longer available for interviews (e.g., moved out of city, phone number no longer in service, or were deceased). A further limitation of the qualitative phase of our study is that we did not pilot-test our interview guides. However, one week before the interviews, participants received an information letter containing examples from the interview questions. Lastly, chiropractors engaged to provide care at the Langs CHC were selected for their focus on evidence-based, time-limited management of musculoskeletal complaints [25, 34]; practice variability among chiropractors in Canada [65] may reduce the generalizability of our findings in other settings.

## Conclusion

We found that patients with chronic non-cancer spinal pain who received chiropractic care obtained fewer and lower dose opioid prescriptions than patients who did not receive chiropractic care. Follow-up interviews suggested this relationship was influenced by patient self-efficacy and concerns about opioid-related harms, limited effectiveness of opioids for chronic pain, stigma regarding use of opioids, and access to non-pharmacological treatment options. Although overall results are promising, large rigorously-conducted randomized controlled trials are needed to establish the role of chiropractic care in reducing opioid use for chronic spinal pain.

## Electronic supplementary material

Below is the link to the electronic supplementary material.

**Additional file 1.** Good Reporting of A Mixed Methods Study (GRAMMS) checklist.
**Additional file 2.** Morphine equivalents daily conversion factors.
**Additional file 3.** Interview guide (patients).
**Additional file 4.** Interview guide (general practitioners).
**Additional file 5.** Investigator reflexivity.
**Additional file 6.** Comparison of recipients versus non-recipients of chiropractic services.
**Additional file 7 (a-g).** Univariable and multivariable regression models for each outcome of interest.
**Additional file 8.** Characteristics of interview participants.
**Additional file 9.** Qualitative themes generated from semi-structured interviews.

## Data Availability

The dataset from this study is held securely in coded form at the Institute for Clinical Evaluative Sciences (ICES). While data sharing agreements prohibit ICES from making the dataset publicly available, access may be granted to those who meet prespecified criteria for confidential access, available at www.ices.on.ca/DAS. The full dataset creation plan and underlying analytic code are available from the corresponding author (PCE) upon reasonable request, understanding that the computer programs may rely upon coding templates or macros that are unique to ICES and are therefore either inaccessible or may require modification.

## List of abbreviations

AIC: Akaike Information Criterion
CHC: Community Health Centre
CI: Confidence Interval
COREQ: Consolidated Criteria for Reporting Qualitative Research
DC: Doctor of Chiropractic
EMR: Electronic Medical Record
GEE: Generalized Estimating Equation
GP: General Practitioner
GRAMMS: Good Reporting of A Mixed Methods Study
ICES: Institute for Clinical Evaluative Sciences
IQR: Inter-quartile range
IRR: Incidence Rate Ratio
MAXQDA: Max Weber Qualitative Data Analysis
MED: Morphine Equivalents Daily
OR: Odds Ratio
QIC: Quasi-likelihood under the Independence model Criterion
SPSS: Statistical Package for the Social Sciences
STROBE: Strengthening the Reporting of Observational Studies in Epidemiology
US: United States
VIF: Variance Inflation Factor
