# Supplementary material for: The association between chiropractic integration in an Ontario community health centre and continued prescription opioid use for chronic non-cancer spinal pain: a sequential explanatory mixed methods study
Source: BMC Health Serv Res. 2022 Nov 3;22:1313. doi: 10.1186/s12913-022-08632-9 (PMC9635131; doi:10.1186/s12913-022-08632-9)
Supplement: Supplementary file 1 — Additional file 1. Good Reporting of A Mixed Methods Study (GRAMMS) checklist. Additional file 2. Morphine equivalents daily conversion factors. Additional file 3. Interview guide (patients). Additional file 4. Interview guide (general practitioners). Additional file 5. Investigator reflexivity. Additional file 6. Comparison of recipients versus non-recipients of chiropractic services. Additional file 7 (a-g). Univariable and multivariable regression models for each outcome of interest. Additional file 8. Characteristics of interview participants. Additional file 9. Qualitative themes generated from semi-structured interviews. [file 12913_2022_8632_MOESM1_ESM.docx]

**Supplementary material**

**Additional file 1:** Good Reporting of A Mixed Methods Study (GRAMMS) checklist

**Additional file 2:** Morphine equivalents daily conversion factors

**Additional file 3:** Interview guide (patients)

**Additional file 4:** Interview guide (general practitioners)

**Additional file 5:** Investigator reflexivity

**Additional file 6:** Comparison of recipients versus non-recipients of chiropractic services

**Additional file 7 (a-g):** Univariable and multivariable regression models for each outcome of interest

**Additional file 8:** Characteristics of interview participants

**Additional file 9:** Qualitative themes generated from semi-structured interviews

**Additional file 1** Checklist of items for the Good Reporting of A Mixed Methods Study (GRAMMS) guidelines [32,33]

| **Mixed methods reporting** | |
| --- | --- |
| **GRAMMS guidelines** | **Location in manuscript where items are reported** |
| 1. Describes the justification for using a mixed methods approach to the research question | Background |
| 1. Describes the design in terms of the purpose, priority and sequence of methods | Methods (Study Design), Figure 1 |
| 1. Describes each method in terms of sampling, data collection and analysis | Methods (Quantitative sampling, Quantitative data collection, Quantitative data analysis, Qualitative sampling, Qualitative data collection, Qualitative data analysis), Figure 1 |
| 1. Describes the integration of the quantitative and qualitative components | Methods (Qualitative sampling, Qualitative data analysis), Results (Qualitative and mixed methods findings), Discussion, Tables 2 and 3, Figure 1 |
| 1. Describes any limitation of one method associated with the presence of the other method | Discussion (Strengths and limitations) |
| 1. Describes any insights gained from mixing or integrating methods | Discussion, Tables 2 and 3 |

*GRAMMS* Good Reporting of A Mixed Methods Study

**Additional file 2** Opioid morphine equivalents daily (MED) conversion factors ^a^ (adapted from Busse et al.[11])

| **Oral opioid formulations** | | | |
| --- | --- | --- | --- |
| ***Opioids*** | | ***Oral MED conversion factors*** | |
| Codeine | | 0.1 to 0.2 | |
| Dihydrocodeine | | 0.1 | |
| Hydrocodone | | 1.0 to 1.5 | |
| Hydromorphone | | 5.0 | |
| Meperidine | | 0.1 | |
| Methadone ^b^ | | 4.0 | |
| Morphine | | 1.0 | |
| Oxycodone | | 1.5 | |
| Oxymorphone | | 3.0 | |
| Tapentadol | | 0.3 to 0.4 | |
| Tramadol | | 0.1 to 0.2 | |
| **Transdermal opioid formulations** | | | |
| ***Opioid*** | ***Hourly microgram dose*** | | ***Mean MED dose (range)*** |
| Fentanyl | 25 mcg/hr | | 97 mg/day (60 to 134) |
| Fentanyl | 37 mcg/hr | | 157 mg/day (135 to 179) |
| Fentanyl | 50 mcg/hr | | 202 mg/day (180 to 224) |
| Fentanyl | 62 mcg/hr | | 247 mg/day (225 to 269) |
| Fentanyl | 75 mcg/hr | | 292 mg/day (270 to 314) |
| Fentanyl | 87 mcg/hr | | 337 mg/day (315 to 359) |
| Fentanyl | 100 mcg/hr | | 382 mg/day (360 to 404) |

*MED* morphine equivalents daily

^a^ These factors are for calculation purposes only

^b^ Oral dose of between 1-20 mg/day

**Additional file 3** Interview guide for patients

**Welcome:**

Introductions and project overview

Before we begin, I would like to review a few items from our consent form:

- Your participation in this project is voluntary and you are free to withdraw from the study (stop the interview completely) at any time.
- Our interview will last about an hour.
- You do not need to answer questions that you do not want to answer or that make you feel uncomfortable.
- All of your answers are private and confidential.
- I will be recording our interview so that all of your ideas are captured.
- You will receive a copy of your interview transcript and a brief summary of the results.

**Introduction:**

Chiropractic services have been available to patients at the Langs Community Health Centre (CHC) since January 1, 2014. I would like to ask you some questions about your experience with the chiropractic services at Langs, as well as whether you feel these services have affected your use of opioids for pain management. I will also ask you for some basic information like your age and formal education. Because the chiropractic program has been put on “pause” during the COVID-19 pandemic, I’d also like to ask you about whether you feel the pandemic, including the reduced access to chiropractic services, has had any additional effect on you with respect to your opioid use.

Are you ready to begin?

1. What year were you born in?
2. How many years have you been a patient at Langs?
3. What is your highest level of education (elementary / high school / college or university / graduate level)?
4. Are you currently working? If so, are you working part- or full-time? If you are *not* working, are you receiving disability benefits / are you retired?
5. Do you currently experience back or neck pain? If **yes**, approximately how long have you had this pain?
6. Are you currently taking any opioid medications for your pain? If so, what opioid medication(s) are you taking (e.g., Tylenol with codeine [Tylenol #3], Percocet or OxyContin [oxycocet, oxycodone], Dilaudid [hydromorphone], Methadose [methadone], Statex or MS Contin [morphine], Tramacet or Ralivia [tramadol], Suboxone [buprenorphine or naloxone], Duragesic [fentanyl patch, or oral fentanyl]) and what is the current dose?

Probe: Has this dose increased, decreased, or stayed the same since you were first prescribed opioids by your general practitioner (GP)?

1. What other types of treatment or activities do you engage in to help manage your pain?
2. Chiropractic services have been offered at Langs since January 1, 2014. Have you ever been referred by your GP for these services? **(If “No,” skip to question #11.)** If **yes**, tell me about your experience with the chiropractic program at Langs?

Probe: Have you found these services helpful? If so, why have you found these services helpful, or if not, why not?

Probe: Had you ever been to a chiropractor before using the chiropractic services here at Langs? If **yes**, do you think this made you more open to being referred by your GP for chiropractic treatment at Langs?

1. Do you feel that the chiropractic services at Langs have had any effect on your opioid use? If so, why do you feel this way, or if not, why not?

Probe: Do you feel you are better able to manage your pain with access to these services?

Probe: Had your GP ever talked to you about reducing your opioid prescriptions and/or doses *before* you were referred for chiropractic treatment? If so, did you agree to work with your GP to reduce your opioid use?

1. The chiropractic program at Langs has been on hold during the COVID-19 pandemic. Has this affected your use of opioids at all? Why or why not?

Probe: If **yes**, how has it affected your use of opioids? Why do you think this is the case, or if not, why not?

Probe: Can you elaborate, or give me any specific examples?

1. **(Skip this question for those who answered “Yes” to question #7.)** If your GP has *never* referred you for chiropractic services at Langs, do you think this type of program would help you with managing your pain and possibly reduce your use of opioids?

Probe: Can you elaborate, or give me any specific examples?

Probe: Have you had any previous experience with chiropractic treatment outside of Langs?

1. Do you think that patients, such as yourself, would benefit from access to pain management services such as chiropractic treatment *before* being prescribed opioids?

Probe: Can you elaborate on why you feel this way, or can you give me any specific examples to help me understand?

1. What advice would you give to another patient who might be considering an opioid prescription to manage their pain?
2. Thank you so much for your time. Do you have any questions, or is there anything else that you would like to share with me?

**Field Notes & Emergent Themes:**

**Additional file 4** Interview guide for general practitioners

**Welcome:**

Introductions and project overview

Before we begin, I would like to review a few items from our consent form:

- Your participation in this project is voluntary and you are free to withdraw from the study (stop the interview completely) at any time.
- Our interview will last approximately one hour.
- You do not need to answer questions that you do not want to answer or that make you feel uncomfortable.
- All of your answers are private and confidential.
- I will be recording our interview to ensure that I accurately capture your statements.
- You will receive a copy of your interview transcript and a summary of the results to confirm I have accurately represented our interview (member-checking).

**Introduction:**

Chiropractic services were integrated at the Langs Community Health Centre (CHC) on January 1, 2014. I would like to ask you some questions about your experience with the chiropractic services at Langs, as well as how you feel these services have affected opioid use among patients at the CHC. I will also ask you for some basic demographic information, such as your age and education. Because the chiropractic program has been put on “pause” during the COVID-19 pandemic, I’d also like to ask you about whether you feel the pandemic, including the reduced access to chiropractic services at Langs, has had any impact on patients’ use of opioids.

Are you ready to begin?

1. In what year were you born?
2. How many years have you been in practice?
3. What is your highest level of education (MD [medical doctor] or NP [nurse practitioner] / other [e.g., MSc or PhD])?
4. Chronic non-cancer pain (CNCP) is any painful condition that persists for at least three months and is not associated with malignant disease. Do you see a lot of patients with CNCP in your practice at Langs?

Probe: For these types of patients, what treatment(s) do you offer or recommend to them?

Probe: What types of medications do you prescribe for pain management?

Probe: For those who have been prescribed opioids, do you feel it would be desirable to have some of these patients reduce their use of opioids?

1. If **not**, why not?
2. If **yes**, have you attempted to engage any of your patients with CNCP in tapering their dose? Or vice versa, have any of these patients asked you about reducing their opioid prescriptions and/or dose?
3. To the best of your knowledge, has there been any opioid-reducing strategy(ies) implemented at Langs to reduce opioid prescribing (e.g., a task force to reduce opioid use, regional dashboards, tracked performance metrics related to high dose prescribing, chart audits, introduction of 2017 CMAJ [Canadian Medical Association Journal] guidelines, etc.)?
4. Chiropractic services have been integrated at Langs since January 1, 2014. Have you ever referred patients for these services?
5. If **not**, why not? **(Then skip to question #7.)**
6. If **yes**, why did you refer patients for these services (e.g., did patients request to be referred for chiropractic treatment, or did you refer them because they were not responding to medical care)?

Probe: Tell me about your experience with the chiropractic program at Langs?

Probe: Have any of your patients found these services helpful? If so, why do you think they have found these services helpful?

Probe: Can you give me any examples of where your patients have *not* found chiropractic treatment to be helpful?

1. Have you ever referred patients for chiropractic care at Langs as part of a formal effort to taper opioids?

Probe: Whether you have or not, what do you think about accessing chiropractic care as part of a strategy to help patients reduce opioid use?

Probe: Was there ever a time where you decided to reduce an opioid dose in a patient you referred for chiropractic services based on the perception that their pain was better managed and thus less opioids were required?

Probe: Do you have any examples of where you referred a patient for chiropractic services but their opioid use stayed the same or increased? If **yes**, can you elaborate on why think this was the case?

1. For this research project, we have been analyzing data on patients with CNCP who were prescribed opioid medication(s) *prior* to being referred for chiropractic treatment. Do you think that patients would be less likely to receive a prescription for opioids if they were referred for chiropractic services *first*? If so why, or if not, why not?

Probe: Can you elaborate, or give me any specific examples?

1. The chiropractic program at Langs has been on hold during the COVID-19 pandemic. How do you feel this has affected opioid use, if at all, among your patients?

Probe: Has this limited the pain management options that you can recommend to your patients?

Probe: Has the pandemic, and lack of access to chiropractic services at Langs, had any impact on the number or dose of opioid medications that you have been prescribing to your patients for pain management?

1. Thank you so much for your time. Do you have any questions, or is there anything else that you would like to share with me on this topic?

**Field Notes & Emergent Themes:**

**Additional file 5** Reporting of investigator reflexivity for the qualitative study phase

| **Research team and reflexivity ^a^** | **Description** |
| --- | --- |
| *Personal characteristics* | |
| Interviewer | All interviews were conducted by the lead author (PCE). |
| Credentials | At the time of the interviews, PCE was a Doctor of Philosophy (PhD) candidate in Health Research Methodology at McMaster University. He also has a Master of Science (MSc) in clinical sciences from Bournemouth University. |
| Occupation | The interviews were coded by two investigators (PCE, ALB). Both are practising Doctors of Chiropractic. PCE is also an adjunct faculty member in the Chiropractic Department at D’Youville University. |
| Gender | PCE identifies as male and ALB identifies as female. |
| Experience and training | PCE has graduate-level training in health research methodology with expertise in mixed methods and qualitative research. PCE and ALB each have over 19 years of clinical and research experience. |
| *Relationship with participants* | |
| Relationship established | PCE worked as a clinician in the chiropractic program at the Langs CHC from January 2014 to January 2016, and therefore had an established relationship with many of the GPs (6/9) and a few of the chiropractic patients (2/8) who were interviewed for this study. These established relationships seemed to facilitate trust and candid conversations during the audio-recorded interviews, particularly with the GPs. PCE did not have a prior relationship with any of the non-chiropractic patients in this study; however, early rapport was developed and established during participant recruitment telephone calls and during introductions and conversation at the start of each interview. |
| Participant knowledge of the interviewer | Participants were sent a form containing information about the study’s aims and objectives, the lead author, and his contact details, at least one week in advance of each interview. Participants were also made aware that the project was being undertaken by PCE as part of a PhD thesis. |
| Interviewer characteristics | Both investigators (PCE, ALB) were interested in the research topic because they had prior experience (PCE: 2 years, ALB: 6 years) working as clinicians in the Langs chiropractic program. This was acknowledged as a potential source for bias in the analysis and interpretation of the data. PCE and ALB each aimed to decrease their own bias throughout the analysis by regularly reflecting on their coding decisions in relation to the study’s aim, and reviewing and recording these reflections at the beginning of each of their peer debriefing meetings. Because of his clinical background as a chiropractor, PCE also practised reflexivity during the course of conducting each interview by maintaining an awareness of how this professional background could bias his assumptions and communication with participants. Accordingly, he made a conscious effort not to stray from the interview guides or ask participants leading questions. |

^a^ Adapted from the Consolidated Criteria for Reporting Qualitative Research (COREQ): <https://academic.oup.com/intqhc/article/19/6/349/1791966>

**Additional file 6** Baseline characteristics of patients from the quantitative chart review (n = 210), stratified by receipt versus non-receipt of chiropractic care

| **Variable ^a^** | **Recipients**  **(n = 49)** | **Non-recipients**  **(n = 161)** | ***P*-value ^b^** |
| --- | --- | --- | --- |
| Age categories, in years   - 18-24 - 25-34 - 35-44 - 45-54 - 55-64 - 65+ | 3 (6.1)  7 (14.3)  6 (12.2)  5 (10.2)  15 (30.6)  13 (26.5) | 2 (1.2)  14 (8.7)  32 (19.9)  44 (27.3)  36 (22.4)  33 (20.5) | 0.028 ^c^ |
| Sex   - Male - Female | 17 (34.7)  32 (65.3) | 72 (44.7)  89 (55.3) | 0.214 |
| General health   - Smoker - Obese ^d^ | 13 (26.5)  6 (12.2) | 63 (39.1)  31 (19.3) | 0.108  0.259 |
| Co-morbidities   - Cardiovascular disease - Depression - Anxiety - Diabetes - Fibromyalgia | 34 (69.4)  30 (61.2)  20 (40.8)  15 (30.6)  8 (16.3) | 102 (63.4)  86 (53.4)  69 (42.9)  45 (28.0)  14 (8.7) | 0.439  0.336  0.800  0.718  0.127 |
| Year of index visit   - 2014 - 2015 - 2016 - 2017 - 2018 - 2019 - 2020 | 32 (65.3)  5 (10.2)  6 (12.2)  4 (8.2)  1 (2.0)  0 (0.0)  1 (2.0) | 87 (54.0)  26 (16.1)  19 (11.8)  8 (5.0)  4 (2.5)  9 (5.6)  8 (5.0) | 0.430 |
| Opioid dosage, median (IQR) | 30 (13.5-56.3) | 30 (15.0-75.0) | 0.487 |

*IQR* inter-quartile range

^a^ Values are expressed as the number (%) unless otherwise noted

^b^ Comparisons between categorical variables or continuous variables were measured using the chi-squared test or the Mann-Whitney U test, respectively

^c^ Statistically significant (2-sided) at an alpha level of 5%

^d^ Patients with a body mass index of ≥ 30 kg/m^2^ were classified as obese

**Additional file 7a** Unadjusted and adjusted incidence rate ratios for the total number of opioid prescription fills over 12 months among patients treated for chronic non-cancer spinal pain between January 1, 2014 and December 31, 2020 (n = 210)

| **Factor** | **Univariable** | ***P*-value** | **Multivariable** | ***P*-value** |
| --- | --- | --- | --- | --- |
|  | **Unadjusted IRR**  **(95% CI)** |  | **Adjusted IRR**  **(95% CI)** |  |
| Chiropractic care   - Exposed (n=49) - Non-exposed (n=161) | 0.69 (0.56-0.85)  Reference | < 0.001 | 0.66 (0.52-0.83)  Reference | < 0.001 |
| Time (calendar year) ^a^ | 1.03 (0.98-1.08) | 0.331 | 1.00 (0.95-1.06) | 0.868 |
| Frequency of healthcare visits ^b^ | 1.00 (0.99-1.01) | 0.847 | 1.01 (0.99-1.02) | 0.300 |
| Age categories   - 18-34 (n=26) ^c^ - 35-44 (n=38) - 45-54 (n=49) - 55-64 (n=51) - 65+ (n=46) | 1.28 (1.02-1.60)  1.30 (1.02-1.64)  1.24 (0.97-1.59)  1.13 (0.90-1.41)  Reference | 0.032  0.031  0.091  0.288 | 1.24 (0.98-1.58)  1.23 (0.95-1.60)  1.13 (0.85-1.51)  1.13 (0.90-1.41)  Reference | 0.075  0.111  0.396  0.284 |
| Sex   - Male (n=89) - Female (n=121) | 0.93 (0.80-1.09)  Reference | 0.372 | 0.92 (0.78-1.08)  Reference | 0.298 |
| Smoking status   - Smoker (n=76) - Non-smoker (n=134) | 0.98 (0.83-1.16)  Reference | 0.820 | 0.92 (0.78-1.07)  Reference | 0.271 |
| Obesity   - Obese (n=37) - Non-obese (n=173) | 1.04 (0.86-1.25)  Reference | 0.716 | 1.04 (0.81-1.35)  Reference | 0.739 |
| Depression   - Present (n=116) - Absent (n=94) | 1.10 (0.94-1.28)  Reference | 0.240 | 1.12 (0.91-1.36)  Reference | 0.282 |
| Anxiety   - Present (n=89) - Absent (n=121) | 1.04 (0.89-1.21)  Reference | 0.653 | 0.98 (0.82-1.18)  Reference | 0.831 |
| Fibromyalgia   - Present (n=22) - Absent (n=188) | 0.96 (0.76-1.22)  Reference | 0.741 | 0.93 (0.72-1.20)  Reference | 0.574 |
| Diabetes   - Present (n=60) - Absent (n=150) | 0.87 (0.74-1.04)  Reference | 0.123 | 0.89 (0.74-1.07)  Reference | 0.212 |
| Cardiovascular disease   - Present (n=136) - Absent (n=74) | 0.94 (0.80-1.09)  Reference | 0.383 | 0.98 (0.82-1.16)  Reference | 0.793 |

*CI* confidence interval, *IRR* incidence rate ratio

^a^ Calendar year was measured at the patient’s index visit date for a non-cancer spine pain diagnosis

^b^ Healthcare visits constitute general practitioner and chiropractic visits

^c^ 18-24 and 25-34 age categories were collapsed and recoded as one category because of a cell frequency of < 10 in the 18-24 group

**Additional file 7b** Unadjusted and adjusted incidence rate ratios for the total number of opioid prescription refills over 12 months among patients treated for chronic non-cancer spinal pain between January 1, 2014 and December 31, 2020 (n = 210) ^a^

| **Factor** | **Univariable** | ***P*-value** | **Multivariable** | ***P*-value** |
| --- | --- | --- | --- | --- |
|  | **Unadjusted IRR**  **(95% CI)** |  | **Adjusted IRR**  **(95% CI)** |  |
| Chiropractic care   - Exposed (n=49) - Non-exposed (n=161) | 0.38 (0.24-0.60)  Reference | < 0.001 | 0.27 (0.17-0.42)  Reference | < 0.001 |
| Time (calendar year) ^a^ | 0.89 (0.76-1.03) | 0.113 | 0.82 (0.73-0.93) | 0.002 |
| Frequency of healthcare visits ^b^ | 1.02 (0.99-1.05) | 0.139 | 1.06 (1.02-1.09) | 0.001 |
| Age categories   - 18-34 (n=26) ^c^ - 35-44 (n=38) - 45-54 (n=49) - 55-64 (n=51) - 65+ (n=46) | 1.15 (0.68-1.95)  1.08 (0.54-2.18)  1.60 (1.04-2.46)  1.20 (0.76-1.90)  Reference | 0.612  0.823  0.031  0.429 | 1.01 (0.59-1.72)  0.88 (0.48-1.61)  1.05 (0.65-1.69)  0.92 (0.60-1.43)  Reference | 0.980  0.684  0.847  0.715 |
| Sex   - Male (n=89) - Female (n=121) | 1.28 (0.91-1.79)  Reference | 0.153 | 1.18 (0.87-1.61)  Reference | 0.281 |
| Smoking status   - Smoker (n=76) - Non-smoker (n=134) | 1.42 (1.01-2.00)  Reference | 0.042 | 1.28 (0.98-1.68)  Reference | 0.075 |
| Obesity   - Obese (n=37) - Non-obese (n=173) | 0.89 (0.61-1.29)  Reference | 0.537 | 0.80 (0.52-1.23)  Reference | 0.302 |
| Depression   - Present (n=116) - Absent (n=94) | 1.15 (0.83-1.60)  Reference | 0.409 | 1.23 (0.83-1.82)  Reference | 0.315 |
| Anxiety   - Present (n=89) - Absent (n=121) | 1.03 (0.73-1.46)  Reference | 0.854 | 0.86 (0.61-1.20)  Reference | 0.360 |
| Fibromyalgia   - Present (n=22) - Absent (n=188) | 1.22 (0.66-2.27)  Reference | 0.529 | 1.22 (0.63-2.34)  Reference | 0.558 |
| Diabetes   - Present (n=60) - Absent (n=150) | 0.98 (0.68-1.43)  Reference | 0.933 | 1.03 (0.72-1.46)  Reference | 0.887 |
| Cardiovascular disease   - Present (n=136) - Absent (n=74) | 1.04 (0.73-1.49)  Reference | 0.826 | 1.10 (0.75-1.61)  Reference | 0.635 |

*CI* confidence interval, *IRR* incidence rate ratio

^a^ Opioid refills were measured in 30-day equivalents

^b^ Calendar year was measured at the patient’s index visit date for a non-cancer spine pain diagnosis

^c^ Healthcare visits constitute general practitioner and chiropractic visits

^d^ 18-24 and 25-34 age categories were collapsed and recoded as one category because of a cell frequency of < 10 in the 18-24 group

**Additional file 7c** Unadjusted and adjusted odds ratios for the likelihood of higher opioid dosage at baseline among patients treated for chronic non-cancer spinal pain between January 1, 2014 and December 31, 2020 (n = 210)

| **Factor** | **Univariable** | ***P*-value** | **Multivariable** | ***P*-value** |
| --- | --- | --- | --- | --- |
|  | **Unadjusted OR**  **(95% CI)** |  | **Adjusted OR**  **(95% CI)** |  |
| Chiropractic care   - Exposed (n=49) - Non-exposed (n=161) | 0.77 (0.38-1.55)  Reference | 0.466 | 0.61 (0.26-1.47)  Reference | 0.270 |
| Time (calendar year) ^a^ | 1.12 (0.95-1.32) | 0.192 | 1.05 (0.88-1.26) | 0.600 |
| Frequency of healthcare visits ^b^ | 1.05 (0.99-1.10) | 0.085 | 1.05 (0.98-1.12) | 0.137 |
| Age categories   - 18-34 (n=26) ^c^ - 35-44 (n=38) - 45-54 (n=49) - 55-64 (n=51) - 65+ (n=46) | 2.08 (0.75-5.76)  1.65 (0.65-4.20)  1.65 (0.68-3.96)  1.07 (0.44-2.64)  Reference | 0.160  0.290  0.266  0.880 | 2.52 (0.84-7.57)  1.92 (0.62-5.93)  1.29 (0.49-3.43)  0.86 (0.35-2.13)  Reference | 0.099  0.255  0.608  0.740 |
| Sex   - Male (n=89) - Female (n=121) | 2.37 (1.31-4.26)  Reference | 0.004 | 2.40 (1.27-4.55)  Reference | 0.007 |
| Smoking status   - Smoker (n=76) - Non-smoker (n=134) | 1.59 (0.88-2.87)  Reference | 0.125 | 1.50 (0.75-3.01)  Reference | 0.254 |
| Obesity   - Obese (n=37) - Non-obese (n=173) | 0.72 (0.33-1.58)  Reference | 0.407 | 0.56 (0.24-1.35)  Reference | 0.197 |
| Depression   - Present (n=116) - Absent (n=94) | 1.29 (0.72-2.31)  Reference | 0.394 | 1.62 (0.78-3.36)  Reference | 0.199 |
| Anxiety   - Present (n=89) - Absent (n=121) | 0.57 (0.31-1.04)  Reference | 0.065 | 0.38 (0.18-0.80)  Reference | 0.011 |
| Fibromyalgia   - Present (n=22) - Absent (n=188) | 0.74 (0.28-1.99)  Reference | 0.557 | 1.16 (0.32-4.21)  Reference | 0.817 |
| Diabetes   - Present (n=60) - Absent (n=150) | 1.03 (0.55-1.95)  Reference | 0.926 | 1.19 (0.57-2.48)  Reference | 0.639 |
| Cardiovascular disease   - Present (n=136) - Absent (n=74) | 1.03 (0.56-1.88)  Reference | 0.923 | 1.37 (0.65-2.89)  Reference | 0.407 |

*CI* confidence interval, *OR* odds ratio

^a^ Calendar year was measured at the patient’s index visit date for a non-cancer spine pain diagnosis

^b^ Healthcare visits constitute general practitioner and chiropractic visits

^c^ 18-24 and 25-34 age categories were collapsed and recoded as one category because of a cell frequency of < 10 in the 18-24 group

**Additional file 7d** Unadjusted and adjusted odds ratios for the likelihood of higher opioid dosage at 3-month follow-up among patients treated for chronic non-cancer spinal pain between January 1, 2014 and December 31, 2020 (n = 210)

| **Factor** | **Univariable** | ***P*-value** | **Multivariable** | ***P*-value** |
| --- | --- | --- | --- | --- |
|  | **Unadjusted OR**  **(95% CI)** |  | **Adjusted OR**  **(95% CI)** |  |
| Chiropractic care   - Exposed (n=49) - Non-exposed (n=161) | 0.42 (0.18-0.99)  Reference | 0.049 | 0.14 (0.04-0.47)  Reference | 0.001 |
| Time (calendar year) ^a^ | 0.89 (0.70-1.12) | 0.308 | 0.73 (0.57-0.94) | 0.015 |
| Frequency of healthcare visits ^b^ | 1.06 (1.00-1.11) | 0.046 | 1.11 (1.02-1.21) | 0.015 |
| Age categories   - 18-34 (n=26) ^c^ - 35-44 (n=38) - 45-54 (n=49) - 55-64 (n=51) - 65+ (n=46) | 1.08 (0.34-3.41)  1.12 (0.40-3.11)  1.75 (0.70-4.38)  1.11 (0.43-2.88)  Reference | 0.896  0.832  0.236  0.834 | 0.86 (0.22-3.38)  0.81 (0.23-2.83)  0.82 (0.28-2.46)  0.61 (0.21-1.74)  Reference | 0.825  0.740  0.727  0.352 |
| Sex   - Male (n=89) - Female (n=121) | 2.17 (1.15-4.09)  Reference | 0.016 | 2.58 (1.24-5.34)  Reference | 0.011 |
| Smoking status   - Smoker (n=76) - Non-smoker (n=134) | 1.67 (0.89-3.15)  Reference | 0.113 | 1.52 (0.73-3.19)  Reference | 0.264 |
| Obesity   - Obese (n=37) - Non-obese (n=173) | 0.52 (0.20-1.32)  Reference | 0.170 | 0.34 (0.12-0.99)  Reference | 0.048 |
| Depression   - Present (n=116) - Absent (n=94) | 1.82 (0.95-3.49)  Reference | 0.069 | 3.29 (1.29-8.35)  Reference | 0.012 |
| Anxiety   - Present (n=89) - Absent (n=121) | 0.77 (0.41-1.46)  Reference | 0.429 | 0.35 (0.13-0.90)  Reference | 0.029 |
| Fibromyalgia   - Present (n=22) - Absent (n=188) | 1.82 (0.72-4.61)  Reference | 0.209 | 3.56 (1.02-12.42)  Reference | 0.047 |
| Diabetes   - Present (n=60) - Absent (n=150) | 1.11 (0.56-2.20)  Reference | 0.763 | 1.36 (0.60-3.12)  Reference | 0.463 |
| Cardiovascular disease   - Present (n=136) - Absent (n=74) | 0.97 (0.50-1.85)  Reference | 0.914 | 1.03 (0.47-2.28)  Reference | 0.936 |

*CI* confidence interval, *OR* odds ratio

^a^ Calendar year was measured at the patient’s index visit date for a non-cancer spine pain diagnosis

^b^ Healthcare visits constitute general practitioner and chiropractic visits

^c^ 18-24 and 25-34 age categories were collapsed and recoded as one category because of a cell frequency of < 10 in the 18-24 group

**Additional file 7e** Unadjusted and adjusted odds ratios for the likelihood of higher opioid dosage at 6-month follow-up among patients treated for chronic non-cancer spinal pain between January 1, 2014 and December 31, 2020 (n = 210)

| **Factor** | **Univariable** | ***P*-value** | **Multivariable** | ***P*-value** |
| --- | --- | --- | --- | --- |
|  | **Unadjusted OR**  **(95% CI)** |  | **Adjusted OR**  **(95% CI)** |  |
| Chiropractic care   - Exposed (n=49) - Non-exposed (n=161) | 0.33 (0.13-0.82)  Reference | 0.018 | 0.14 (0.05-0.40)  Reference | < 0.001 |
| Time (calendar year) ^a^ | 0.94 (0.76-1.16) | 0.573 | 0.78 (0.62-0.99) | 0.041 |
| Frequency of healthcare visits ^b^ | 1.04 (0.99-1.10) | 0.137 | 1.09 (1.01-1.18) | 0.027 |
| Age categories   - 18-34 (n=26) ^c^ - 35-44 (n=38) - 45-54 (n=49) - 55-64 (n=51) - 65+ (n=46) | 1.26 (0.44-3.64)  0.88 (0.33-2.38)  1.25 (0.51-3.06)  0.69 (0.27-1.80)  Reference | 0.670  0.800  0.625  0.448 | 1.13 (0.28-4.52)  0.65 (0.20-2.04)  0.59 (0.21-1.71)  0.38 (0.13-1.13)  Reference | 0.863  0.456  0.335  0.081 |
| Sex   - Male (n=89) - Female (n=121) | 2.06 (1.10-3.85)  Reference | 0.024 | 2.46 (1.21-4.96)  Reference | 0.012 |
| Smoking status   - Smoker (n=76) - Non-smoker (n=134) | 1.60 (0.85-3.01)  Reference | 0.145 | 1.42 (0.67-3.01)  Reference | 0.358 |
| Obesity   - Obese (n=37) - Non-obese (n=173) | 0.91 (0.40-2.09)  Reference | 0.831 | 0.67 (0.25-1.85)  Reference | 0.443 |
| Depression   - Present (n=116) - Absent (n=94) | 1.90 (0.99-3.63)  Reference | 0.052 | 2.67 (1.14-6.26)  Reference | 0.023 |
| Anxiety   - Present (n=89) - Absent (n=121) | 1.01 (0.54-1.89)  Reference | 0.971 | 0.55 (0.24-1.22)  Reference | 0.141 |
| Fibromyalgia   - Present (n=22) - Absent (n=188) | 1.40 (0.54-3.64)  Reference | 0.490 | 2.24 (0.70-7.16)  Reference | 0.174 |
| Diabetes   - Present (n=60) - Absent (n=150) | 1.07 (0.54-2.12)  Reference | 0.842 | 1.14 (0.51-2.56)  Reference | 0.758 |
| Cardiovascular disease   - Present (n=136) - Absent (n=74) | 1.25 (0.65-2.43)  Reference | 0.503 | 1.36 (0.62-2.99)  Reference | 0.446 |

*CI* confidence interval, *OR* odds ratio

^a^ Calendar year was measured at the patient’s index visit date for a non-cancer spine pain diagnosis

^b^ Healthcare visits constitute general practitioner and chiropractic visits

^c^ 18-24 and 25-34 age categories were collapsed and recoded as one category because of a cell frequency of < 10 in the 18-24 group

**Additional file 7f** Unadjusted and adjusted odds ratios for the likelihood of higher opioid dosage at 9-month follow-up among patients treated for chronic non-cancer spinal pain between January 1, 2014 and December 31, 2020 (n = 210)

| **Factor** | **Univariable** | ***P*-value** | **Multivariable** | ***P*-value** |
| --- | --- | --- | --- | --- |
|  | **Unadjusted OR**  **(95% CI)** |  | **Adjusted OR**  **(95% CI)** |  |
| Chiropractic care   - Exposed (n=49) - Non-exposed (n=161) | 0.39 (0.17-0.94)  Reference | 0.035 | 0.19 (0.07-0.57)  Reference | 0.003 |
| Time (calendar year) ^a^ | 1.01 (0.83-1.23) | 0.918 | 0.88 (0.71-1.09) | 0.234 |
| Frequency of healthcare visits ^b^ | 1.04 (0.99-1.09) | 0.129 | 1.10 (1.02-1.19) | 0.018 |
| Age categories   - 18-34 (n=26) ^c^ - 35-44 (n=38) - 45-54 (n=49) - 55-64 (n=51) - 65+ (n=46) | 0.84 (0.29-2.46)  0.71 (0.27-1.88)  1.01 (0.42-2.42)  0.56 (0.22-1.42)  Reference | 0.753  0.491  0.985  0.220 | 0.63 (0.18-2.18)  0.47 (0.15-1.45)  0.50 (0.18-1.42)  0.32 (0.11-0.87)  Reference | 0.469  0.189  0.194  0.026 |
| Sex   - Male (n=89) - Female (n=121) | 1.95 (1.05-3.64)  Reference | 0.035 | 1.63 (0.81-3.31)  Reference | 0.173 |
| Smoking status   - Smoker (n=76) - Non-smoker (n=134) | 1.25 (0.66-2.35)  Reference | 0.494 | 1.41 (0.67-3.00)  Reference | 0.367 |
| Obesity   - Obese (n=37) - Non-obese (n=173) | 0.89 (0.39-2.02)  Reference | 0.776 | 0.90 (0.31-2.64)  Reference | 0.848 |
| Depression   - Present (n=116) - Absent (n=94) | 1.30 (0.70-2.43)  Reference | 0.409 | 2.16 (0.94-4.98)  Reference | 0.070 |
| Anxiety   - Present (n=89) - Absent (n=121) | 0.58 (0.30-1.10)  Reference | 0.094 | 0.34 (0.15-0.78)  Reference | 0.010 |
| Fibromyalgia   - Present (n=22) - Absent (n=188) | 0.81 (0.29-2.32)  Reference | 0.697 | 1.35 (0.20-2.78)  Reference | 0.654 |
| Diabetes   - Present (n=60) - Absent (n=150) | 1.04 (0.53-2.04)  Reference | 0.921 | 0.82 (0.36-5.11)  Reference | 0.626 |
| Cardiovascular disease   - Present (n=136) - Absent (n=74) | 0.76 (0.40-1.43)  Reference | 0.390 | 0.69 (0.32-1.47)  Reference | 0.333 |

*CI* confidence interval, *OR* odds ratio

^a^ Calendar year was measured at the patient’s index visit date for a non-cancer spine pain diagnosis

^b^ Healthcare visits constitute general practitioner and chiropractic visits

^c^ 18-24 and 25-34 age categories were collapsed and recoded as one category because of a cell frequency of < 10 in the 18-24 group

**Additional file 7g** Unadjusted and adjusted odds ratios for the likelihood of higher opioid dosage at 12-month follow-up among patients treated for chronic non-cancer spinal pain between January 1, 2014 and December 31, 2020 (n = 210)

| **Factor** | **Univariable** | ***P*-value** | **Multivariable** | ***P*-value** |
| --- | --- | --- | --- | --- |
|  | **Unadjusted OR**  **(95% CI)** |  | **Adjusted OR**  **(95% CI)** |  |
| Chiropractic care   - Exposed (n=49) - Non-exposed (n=161) | 0.52 (0.23-1.19)  Reference | 0.123 | 0.22 (0.08-0.62)  Reference | 0.004 |
| Time (calendar year) ^a^ | 0.96 (0.78-1.19) | 0.705 | 0.82 (0.66-1.04) | 0.097 |
| Frequency of healthcare visits ^b^ | 1.06 (1.01-1.12) | 0.018 | 1.12 (1.03-1.21) | 0.006 |
| Age categories   - 18-34 (n=26) ^c^ - 35-44 (n=38) - 45-54 (n=49) - 55-64 (n=51) - 65+ (n=46) | 1.04 (0.35-3.10)  0.76 (0.27-2.10)  1.25 (0.51-3.06)  0.69 (0.27-1.80)  Reference | 0.938  0.590  0.625  0.448 | 0.69 (0.19-2.55)  0.54 (0.17-1.78)  0.62 (0.24-1.64)  0.46 (0.16-1.31)  Reference | 0.582  0.313  0.336  0.146 |
| Sex   - Male (n=89) - Female (n=121) | 1.36 (0.72-2.55)  Reference | 0.339 | 1.22 (0.60-2.49)  Reference | 0.591 |
| Smoking status   - Smoker (n=76) - Non-smoker (n=134) | 1.14 (0.60-2.17)  Reference | 0.694 | 1.16 (0.55-2.44)  Reference | 0.692 |
| Obesity   - Obese (n=37) - Non-obese (n=173) | 0.42 (0.15-1.14)  Reference | 0.088 | 0.38 (0.12-1.22)  Reference | 0.103 |
| Depression   - Present (n=116) - Absent (n=94) | 1.57 (0.82-2.99)  Reference | 0.171 | 2.27 (0.99-5.24)  Reference | 0.054 |
| Anxiety   - Present (n=89) - Absent (n=121) | 0.90 (0.47-1.70)  Reference | 0.737 | 0.58 (0.26-1.29)  Reference | 0.179 |
| Fibromyalgia   - Present (n=22) - Absent (n=188) | 1.16 (0.43-3.13)  Reference | 0.773 | 1.35 (0.39-4.69)  Reference | 0.633 |
| Diabetes   - Present (n=60) - Absent (n=150) | 0.79 (0.39-1.61)  Reference | 0.512 | 0.94 (0.42-2.12)  Reference | 0.880 |
| Cardiovascular disease   - Present (n=136) - Absent (n=74) | 0.83 (0.43-1.59)  Reference | 0.575 | 0.85 (0.40-1.81)  Reference | 0.669 |

*CI* confidence interval, *OR* odds ratio

^a^ Calendar year was measured at the patient’s index visit date for a non-cancer spine pain diagnosis

^b^ Healthcare visits constitute general practitioner and chiropractic visits

^c^ 18-24 and 25-34 age categories were collapsed and recoded as one category because of a cell frequency of < 10 in the 18-24 group

**Additional file 8** Demographic and clinical characteristics of participants from the qualitative interviews (n = 23)

| **Variable** | **Value ^a^** | |
| --- | --- | --- |
|  | **Patients (n = 14) ^b^** | **GPs (n = 9) ^b^** |
| Age in years, mean (SD) | 56.2 (14.3) | 47 (10.5) |
| Sex   - Male - Female | 3 (21.4)  11 (78.6) | 2 (22.2)  7 (77.8) |
| Years attending CHC (patients) / years in practice (GPs), mean (SD) | 13 (11.6) | 13.4 (6.8) |
| Completed post-secondary education or higher | 7 (50.0) | 9 (100) |
| Receiving disability benefits / unemployed | 12 (85.7) | 0 (0) |
| Opioid prescription | 10 (71.4) | NA |
| Receipt of chiropractic care | 8 (57.1) | NA |

*CHC* community health centre, *GP* general practitioner, *NA* not applicable, *SD* standard deviation

^a^ Values are expressed as the number (%) unless otherwise noted

^b^ Non-participants were similar to participants in terms of age, sex, years attending the CHC (patients) or years in practice (GPs), exposure to chiropractic care, and opioid prescription

**Additional file 9** Qualitative themes generated from semi-structured interviews with patients (n = 14) and general practitioners (n = 9) at the Langs Community Health Centre regarding perceptions of chiropractic integration and its impact on opioid prescribing

| **Themes ^a^** | **Description** | **Sub-themes and representative quotes ^b^** |
| --- | --- | --- |
| 1. Patient self-efficacy   (n = 23) | Patients and GPs described active and passive pain management strategies and behaviours, including a resistance among some patients toward taking medication. | Active versus passive approaches:   - *“****I’m continuously doing stretches, exercises, to keep myself okay.****”* DC Patient 2 - *“****I try exercising, but it just makes it worse.****”* DC Patient 7 - *“It’s really* ***hard to engage some of these folks in self-care*** *with their chronic pain. You know? It’s just like* ***– give me medication.****”* GP 1 - *“****A lot of our folks don’t feel very empowered, or don’t feel like they have much agency in their lives****, which is true. … With chronic back [pain], … probably the greatest utility [of an active approach] is having them* ***understand that some of this is within their control to influence.****”* GP 7 - *“[For a patient who might be considering an opioid prescription to manage their pain], I would just say to* ***start trying to feel some of your pain again so that you can learn how to manage it in a different way.*** *You know?* ***Instead of just,*** *like,* ***burying all the pain.****”* Non-DC Patient 5 - *“When [patients] come in believing that you can find the right thing that will fix their pain, and – opioid medication is one component of that belief system – I think that the more we engage in that from the get-go, the less successful we’re going to be at changing that mindset. … Passive therapies such as opioids, or even chiropractic manipulation or massage [by themselves], are not necessarily bad; but* ***if [passive therapy] is our starting point*** *– and again, this is just what I’ve experienced – [I feel]* ***it is harder to engage patients in active options later.****”* GP 4   Resistance to taking medication:   - ***“…most patients don’t want to be on a bunch of pills. They don’t want to be dependent on medication to feel better.****”* GP 9 - *“I’ve been dealing with this pain for 10 years, and* ***I’m not just a pill popping, believing [person].*** *[I’m] old school, take the pain until it’s really extreme and then – oh gee, I better take an Advil – is kinda how I deal with my pain.”* DC Patient 1 - *“****Most people don’t want to take pills*** *from my perspective – for a multitude of reasons.* ***They prefer to have all the options for treatment****, and that includes non-pharm[acological] solutions.”* GP 2 |
| 1. Accessibility of non-pharmacological services   (n = 21) | Lack of access to non-pharmacological services was identified as a common facilitator of opioid use. | Lack of access to non-pharmacological treatment options:   - *“Most of our patients don’t have any additional coverage for extended health, like, physio, chiro, all of those things. So, unfortunately,* ***even though those are first-line modalities for treating a lot of pain, patients can’t access it unless we have programs for it.****”* GP 8 - *“It’s hard when you have nothing to give them.* ***I think that’s why, in part, what’s driven opioid over-prescribing is because we have nothing else to give these people.*** *… I think* ***having access to any kind of additional modalities in a timely and efficient manner*** *for all patients* ***would probably reduce the need for opioids in the first place.****”* GP 9 - *“****The government needs to step up and help out.****”* DC Patient 3 - *“It’s that scenario where you have nothing else to offer, right? So, if you’re trying to postpone heading into ‘opioid land’ and you still have something else to offer, it can definitely make a difference. …* ***You probably go to meds sooner than you might otherwise because you don’t have access to the intervention you’d really like.****”* GP 7 - *“When I was about 23 [years old],* ***financially I wasn’t able to go [to my chiropractor] anymore. So, that’s when [my doctor] put me on the OxyContin and the Perc’s.****”* DC Patient 7 - *“We’re told there’s a triangle of care, you know, the psychosocial, and the physical modalities, and meds, [which] are only a small part of it. But the only thing you have access to is the meds. …* ***It’s just ironic. The people who need [non-pharmacological] services the least, have the best access.*** *…* ***But the people who are most vulnerable to [chronic pain] are the people that have the least access.****”* GP 9   Access to chiropractic services at the Langs CHC:   - *“The [chiropractic] program at Langs was helpful in that,* ***it allowed more access for people that didn’t have it otherwise.****”* GP 9 - *“****The evidence says you guys are as good at clearing up back pain as we are.*** *So, we need to work together at that, and it’s helpful to have [access to chiropractic services for our patients].”* GP 3 - *“****The folks we take care of, 95% of them couldn’t afford chiropractic on their own, or don’t have [a] benefit plan.*** *There’s a small portion who do, but most of them don’t. So, being able to, right from the outset, present this package of care, I think [is] incredibly helpful.”* GP 7 - *“****I’m missing the chiropractor services*** *[since they were discontinued because of the COVID-19 pandemic]. I wish it could come back again, because I’m having pain.”* DC Patient 2 |
| 1. Stigma regarding use of prescription opioids   (n = 20) | A negative stigma around opioids was identified by patients and GPs as a barrier to opioid use. However, patients who found benefit in using opioids often felt a sense of judgement, as well as a need to advocate for themselves to obtain an opioid prescription. | - *“I think* ***in our current society with news about addiction*** *and our history of OxyContin over-prescribing,* ***we probably have fewer people asking for opioids.****”* GP 3 - *“****Sometimes we have people we can’t even talk into considering an opioid*** *because they’ve taken what they’ve heard in the media so significantly they don’t want anything to do with that.”* GP 7 - *“Unfortunately,* ***patients experience a great deal of stigma****, and so some of them don’t like that. And then they want to come off of their pills.”* GP 9 - *“I take … the Tylenol #3, [but] it’s no good,* ***it’s [like] cocaine.****”* DC Patient 4 - *“****It’s been frustrating – so frustrating. Because the [opioid] crisis seemed to just fall right on me.*** *Like, as though I’m part of the crisis. So, [as a result] every doctor doesn’t want you on any kind of pain medication. They don’t believe your pain. You know what I mean? It has really affected me. …* ***I’m not an addict in any way.*** *I never even ever think twice about taking that medication more than once, like, unprescribed. But I was definitely treated like I was [an addict].”* Non-DC Patient 5 |
| 1. Impact of treatment   (n = 19) | Patients and GPs described their impressions of treatment directed at managing chronic pain. | Pain relief:   - *“[For] my neck, sometimes,* ***if I didn’t go [to the chiropractor], I would really notice it*** *in a couple months if I didn’t go every, at least every two months, if not every month.”* DC Patient 8 - *“When you have a nerve condition, especially like mine where the nerves are hypersensitive and will shoot off through your entire body like electricity, sometimes with, you know, airflow. … It’s literally, like, I’ve been outside and a strong wind has gone across my legs and it makes my, it just makes my heart start pounding so much because it hurts that much and I’m like – are you freaking kidding me? And in that situation, it’s just like – no,* ***I think I actually need something very, very strong to calm these nerves down or to numb my body or these nerves.****”* Non-DC Patient 1 - *“I feel like it’s more a push from the patient to do something about [their] pain and to help [them]. … Because* ***ultimately, you’re trying to alleviate their suffering.*** *That’s why they come. And, you’re trying to respond to that.”* GP 9   Functionality:   - *“I know that* ***I would not be able to function without having some relief [from opioids].****”* Non-DC Patient 4 - *“****I do actually have patients on opioids that are actually working and it’s because they’re on opioids****, … that they continue to work full-time. And so, they’re not the ones that I worry about so much because* ***they clearly have functionality****, and they don’t show any behavioural stuff.”* GP 9   Recognition of the limited effectiveness of opioids for chronic pain:   - *“In my experience****, the medication [Percocet] does not take the pain away.*** *It will dull the pain so you can function, but it won’t take it away. … And that’s on good days. Sometimes if it’s a rough day, then even the medication is not very helpful.”* Non-DC Patient 4 - *“[I’ve been taking pain medications since] 2012. … It never feel[s] good.* ***[Even though] I take three [Tylenol #3] pills every day, … I still have a lot of pain.****”* DC Patient 4 - *“I’m glad I’m off of the fentanyl, [but]* ***the Butran [buprenorphine] is not holdin’ all my pain.*** *You know?* ***It’s just takin’ a little bit [of it away].****”* Non-DC Patient 6   Fear or anxiety of withdrawal:   - *“****There’s a lot of fear around it*** *of – if they are managed well with their opioids – that they might go back to a situation where they’re feeling a lot of pain and not functioning properly.”* GP 5 - *“…****terrifying.*** *… Not being able to have [my] prescription filled is* ***very frightening – and panic. You start having anxiety.****”* Non-DC Patient 4 - *“I think sometimes with the patients on opioids* ***there’s a lot of fear and anxiety about the dosing and the reduction of the dose because it’s unpleasant.*** *And because they’re afraid about their pain and stuff like that.”* GP 1 - *“****I was feeling more [pain] right away*** *within the first time that I went down [in my opioid dose]. That was from the 6’s to the 3’s [6 mg of Hydromorphone to 3 mg], twice a day.* ***And I remember that, and thinking –*** *oh my goodness,* ***I’m going to go back to square one*** *if they keep doing this. Which, I didn’t want to be in that much pain.”* Non-DC Patient 5 - *“I have to be honest,* ***people are so anxious when you’re talking about decreasing their opioids, they don’t hear much else.****”* GP 7 |

*CHC* community health centre, *DC* doctor of chiropractic, *GP* general practitioner

^a^ Among all 23 interview participants, 19 touched upon each of the four major themes. Of the remaining four participants, two touched upon two of the four themes while the other two touched upon three of the four themes

^b^ Bolded phrases are for thematic emphasis
